# Pathology-Anchored Biomarker Research Progress for the Early Diagnosis of Diabetic Kidney Disease: From Pathological Association to Early Validation

**DOI:** 10.3390/biomedicines14071643

**Published:** 2026-07-21

**Authors:** Qiu Li, Mei Yang, Yingyu Luo, Nannan Zhang

**Affiliations:** 1 Department of Nephrology, The First People’s Hospital of Shuangliu District, Chengdu 610299, China; sllq429@163.com (Q.L.); ym135688@163.com (M.Y.); 2 National Center for Birth Defect Monitoring, West China Second University Hospital, Sichuan University, Chengdu 610041, China; 17844599731@163.com; 3 Key Laboratory of Birth Defects and Related Diseases of Women and Children (Sichuan University), Ministry of Education, Chengdu 610041, China

**Keywords:** diabetic kidney disease, pathology-anchored, biomarkers, multi-omics, early diagnosis

## Abstract

Diabetic kidney disease (DKD) is a leading cause of end-stage renal disease (ESRD). Early diagnosis is therefore critical for improving patient outcomes.However, traditional clinical biomarkers are constrained by diagnostic latency and limited sensitivity, particularly in cases of non-albuminuric DKD. To address these limitations, this review systematically explores the technical framework of the pathology-anchored strategy and proposes a two-phase translational approach, consisting of Pathology Anchoring Discovery and Prospective Early Validation. This strategy employs renal biopsy as the pathological gold standard in conjunction with multi-omics technologies to correlate circulating or urinary molecules with specific renal histological lesions, ultimately identifying non-invasive biomarkers with definitive pathological relevance. While numerous biomarkers demonstrate early warning potential in high-risk populations with normal conventional indicators, the pathology-anchored framework serves as a critical bridge linking these clinical biomarkers and distinct pathological changes. This review presents potential insights for early identification, risk stratification, and prognostic assessment of DKD.

## 1. Introduction

Diabetic kidney disease (DKD) has emerged as the predominant cause of end-stage renal disease (ESRD) globally. It is estimated that approximately 30% of individuals with type 1 diabetes and around 40% of those with type 2 diabetes develop DKD over the course of their illness, thereby creating a massive healthcare burden [1,2]. While early intervention can markedly slow the disease progression, current clinical diagnostic practices still predominantly rely on the urinary albumin-to-creatinine ratio (UACR) and the estimated glomerular filtration rate (eGFR). Unfortunately, these traditional markers present several critical limitations, characterized by diagnostic latency and poor sensitivity, which fail to identify non-albuminuric DKD [3]. Furthermore, individuals with type 2 diabetes may concurrently suffer from primary glomerular diseases, and clinical indicators alone are insufficient to accurately differentiate between DKD and non-DKD [4,5]. Consequently, there is an urgent need for novel biomarkers to facilitate the early diagnosis of DKD [4,6,7].

Previous research on DKD biomarkers has predominantly focused on association analyses with clinical phenotypes by merely comparing serum or urine molecular levels between patients and controls, often lacking histopathological validation to confirm their renal tissue origin. Consequently, the relationship between these biomarkers and disease mechanisms remains ambiguous, limiting their diagnostic efficacy [8,9]. However, renal biopsy is not feasible for all diabetic patients due to its invasive nature, potential complications, and strict clinical criteria [10,11].

**Figure 1:**
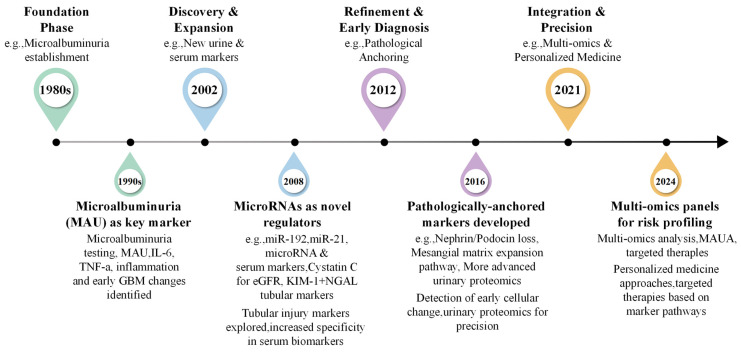
Schematic diagram of the development status of novel DKD biomarkers based on pathology anchoring. Timeline of key milestones in diabetic kidney disease biomarkers from the 1980s to 2024, including microalbuminuria, inflammatory cytokines, microRNAs, tubular injury markers, pathological anchoring, urinary proteomics, and multi-omics integration; MAU, microalbuminuria; miR, microRNA; eGFR, estimated glomerular filtration rate; KIM-1, kidney injury molecule-1; NGAL, neutrophil gelatinase-associated lipocalin.

Innovative approaches are therefore required to integrate pathological insights into non-invasive testing. For instance, a recent Australian study developed an immunoassay-based test that integrates plasma protein biomarkers with clinical variables to predict the risk of renal function decline in patients with type 2 diabetes [12]. Building upon such efforts to address the insufficient pathological specificity of traditional noninvasive biomarkers, the “pathology-anchored” research strategy has been developed. Utilizing kidney biopsy pathology as the gold standard, this approach employs high-throughput omics technologies, including transcriptomics, proteomics, and metabolomics, to accurately correlate circulating or urinary molecular biomarkers with specific renal tissue lesions in patients with DKD, such as podocyte injury, endothelial proliferation, tubulointerstitial fibrosis, and inflammatory cell infiltration. This methodology facilitates the identification of noninvasive biomarkers that possess distinct pathological relevance [13,14]. The timeline of the development of biomarkers for DKD has been summarized in Figure 1.

This review systematically explores the pathology-anchored research strategy and proposes a two-phase translational approach. By highlighting the integration of the discovery and validation stage, this design provides a complementary perspective to previous biomarker reviews. Ultimately, this framework could serve as a promising theoretical route to facilitate pathology-guided early DKD diagnosis and risk stratification.

Published studies exploring pathology-related biomarkers for the early diagnosis of diabetic kidney disease were retrieved from PubMed and Web of Science. The main search keywords included diabetic kidney disease, early diagnosis, renal pathology and biomarker. Boolean operators including AND and OR were used to combine search terms. We prioritized high-quality original studies and authoritative reviews published in recent years, and selected eligible studies based on their tight relevance to the topic of pathological correlation and early clinical validation of biomarkers.

## 2. Technological Basis for the Discovery of Pathological-Anchored Biomarkers

### 2.1. A Two-Stage Design from Pathological Association to Early Validation

Due to ethical restrictions, kidney biopsy tissues cannot be directly obtained from diabetic patients whose traditional indicators (eGFR and UACR) are within normal ranges. Consequently, the concept of “pathology-anchored early diagnosis” does not imply performing renal punctures on patients in the early stages of the disease. Instead, it employs a two-stage inferential design.

Stage 1 Pathology Anchoring Discovery

Clinically indicated kidney biopsy specimens from patients with DKD, characterized by proteinuria or a decline in eGFR, serve as the pathological reference for screening lesion-anchored non-invasive biomarkers. This approach parallels the oncology paradigm, wherein tissue biomarkers from advanced tumors, such as Septin9 methylation in colorectal cancer, are validated for early detection in asymptomatic populations [8]. A similar rationale applies to DKD, as biopsies cannot ethically be performed on normoalbuminuric individuals without clinical indications [10,11,15]. Therefore, clinically indicated biopsies represent the only viable pathological anchor, necessitating a second stage of prospective validation in high-risk normoalbuminuric cohorts.

Stage 2 Prospective Early Validation

Candidate molecules are evaluated within independent prospective cohorts of diabetic patients who exhibit normal eGFR and UACR, yet possess elevated risk factors. This evaluation aims to ascertain whether these molecules can predict the onset of disease prior to the manifestation of abnormalities in conventional indicators [4,16]. Concrete examples include urinary nephrin mRNA, which serves as a predictor for the progression to microalbuminuria in individuals with normoalbuminuric type 2 diabetes [16], and multi-marker panels such as MMP-7/SAA1/TNC (matrix metalloproteinase 7, serum amyloid A1, tenascin C), which have demonstrated similar prospective validation [17]. These studies illustrate how specific molecules associated with distinct renal lesions, such as podocyte injury and matrix remodeling, are validated through longitudinal follow-up and histopathological scoring. This pathology-anchored approach is based on a comprehensive understanding of DKD pathogenesis, which involves hemodynamic, metabolic, inflammatory, oxidative, and fibrotic pathways [18,19]. Chronic hyperglycemia induces pericyte apoptosis and microvascular rarefaction [20], while lipotoxicity exacerbates damage to podocytes and tubular cells through inflammation and cell death pathways [21]. Therefore, non-invasive biomarkers must be linked to these well-defined pathological processes-podocyte injury, tubulointerstitial fibrosis, and inflammatory infiltration-before they can be integrated into the anchoring system [22]. While kidney biopsy remains the definitive diagnostic tool, its invasive nature is noteworthy. Large-scale, multicenter data from the German DeGIR registry, encompassing 5235 procedures, indicate a severe complication rate of 0.74% [10]. Additionally, obtaining an insufficient number of glomeruli (fewer than 10) can compromise the accuracy of pathological grading [11]. Despite these limitations, the biopsy continues to play a pivotal role. However, there is a need for standardized scoring systems, such as the Tervaert classification, and multicenter quality control to mitigate anchoring errors in translational studies [11]. Ultimately, the primary advantage of this approach lies in enhancing the specificity of biomarkers by spatially correlating molecular signals with in situ renal histopathology, thereby improving diagnostic precision and mechanistic understanding [22].

Nevertheless, the two-stage inferential design is predicated on a crucial assumption: that the expression profiles of molecular biomarkers identified in kidney tissues affected by advanced DKD are consistent with the pathological changes occurring in the early subclinical stage. This assumption, however, has not been comprehensively validated. The pathophysiological mechanisms underlying DKD are dynamic, evolving from early to late stages, and encompass multiple axes, including hemodynamic alterations, metabolic disturbances, inflammation, and fibrosis [18]. Consequently, molecular expression profiles may vary substantially between early and late stages. Certain genes, particularly those associated with immune and fibrosis-related pathways, which are highly upregulated in the late stages of the disease, may exhibit no significant change or may even be downregulated during the early stages. In contrast, adaptive signals that are amplified during the early stages of disease may be overshadowed by inflammation and fibrosis in the later stages.

### 2.2. Frontier Methods Required for Implementing Pathology Anchoring

#### 2.2.1. Spatial Omics Technologies

Spatial omics and body-fluid omics technologies serve as crucial links between molecular characteristics and pathological phenotypes. Spatial transcriptomics offers precise mapping of gene expression to specific pathological areas, such as glomerulosclerosis and interstitial fibrosis, thereby elucidating their in situ molecular attributes [17,23]. Advanced techniques like imaging mass cytometry and matrix-assisted laser desorption/ionization mass spectrometry imaging facilitate the colocalization of cell subpopulations with pathological injuries at the protein level. These methods have been instrumental in differentiating protein profiles across various kidney disease types [24]. Additionally, body-fluid analysis technologies, including urinary proteomics, enable the identification of noninvasive biomarkers with potential differential diagnostic value, such as Serpin A4 and gamma-glutamyltransferase 1 (GGT1), by comparing protein profiles between DKD and non-diabetic kidney disease [25]. Collectively, these technologies provide a comprehensive research toolkit, spanning from mechanistic insights to clinical applications, for the discovery of DKD biomarkers with spatial or diagnostic specificity [26].

These technologies, while primarily utilized as research tools, have demonstrated significant value in pathology. Zhou et al. employed spatial transcriptomics (10× Visium) in conjunction with single-cell RNA sequencing to examine kidney tissues from patients with DKD [17]. Their analysis revealed a significant elevation of matrix metalloproteinase 7 (MMP 7) and Tenascin C (TNC) in sclerotic glomeruli and fibrotic interstitium. Notably, spatial transcriptomics identified 13 differentially expressed genes within the glomerular region, including MMP 7, TNC, Collagen type I alpha 1 (COL1A1), Collagen type I alpha 2 (COL1A2), fibronectin 1(FN1), Lumican (LUM), versican (VCAN), among others, as well as a distinct expression signature in the fibrotic interstitium. Utilizing serum proteomics and machine learning, the study developed a diagnostic model that incorporated three protein biomarkers (MMP 7, SAA1, and TNC) alongside a serum metabolite signature (the specific metabolites were not detailed in the original study). This model enhanced the accuracy of early DKD detection by approximately 13%, differentiated between early and advanced disease progression, and was validated across four independent clinical cohorts and multiple organs in db/db mice [17]. These findings collectively demonstrate a closed-loop translational pathway that integrates spatial and single-cell omics with noninvasive serum biomarkers. In a recent study, Dumoulin et al. employed spatial transcriptomics and single-cell sequencing to develop a high-resolution spatial atlas of DKD kidneys, identifying a distinct disease subgroup characterized by B-cell enrichment [27]. This research unveiled previously unrecognized immune pathological heterogeneity within DKD and identified chemokine ligands, such as CXCL13, as well as potential autoantigens associated with regions of B-cell infiltration. These findings offer novel targets for the development of pathology-anchored noninvasive biomarkers aimed at specific immune cell subpopulations, including urinary B-cell-related chemokines or plasma cell-derived immunoglobulins, thereby extending the applicability of spatial omics in DKD pathology-focused research.

#### 2.2.2. Single-Cell and Single-Nucleus Transcriptomics

Single-cell RNA sequencing (scRNA-seq) offers a powerful tool for elucidating the heterogeneity of intrinsic renal cells and identifying cell-subpopulation-specific biomarkers [8]. This approach often involves the utilization of animal models, such as genetically modified rodent models of diabetic nephropathy, to replicate specific pathological characteristics of human DKD [28]. By integrating scRNA-seq, researchers can pinpoint the expression and functional roles of biomarkers within distinct renal cell types, including proximal tubular cells and podocytes [13]. The human kidney single-cell atlas, developed by Lake et al., has unveiled transcriptomic alterations across various cell subpopulations under DKD conditions, thereby providing a crucial resource for the identification of pathology-anchored biomarkers [13]. Recent investigations employing scRNA-seq have demonstrated that elevated tripartite motif-containing protein 22 (TRIM22) expression in renal tubular epithelial cells is significantly correlated with autophagy dysregulation and the extent of interstitial fibrosis. Following validation through immunohistochemistry, TRIM22 emerges as a potential biomarker for tubulointerstitial injury [6]. Single-nucleus RNA sequencing (snRNA-seq) has facilitated the extension of analyses to archived frozen tissues. A notable study utilizing this technique identified dysregulated expression of genes associated with neutrophil extracellular traps, such as ITGAM and ITGB2, in DKD tissues. These findings were corroborated by corresponding protein alterations observed in patient blood samples, indicating that these molecules may serve as indicators of immune-related pathological processes [29]. Furthermore, the integration of spatial transcriptomics with scRNA-seq has advanced the ability to localize molecular alterations to specific histological lesions. For instance, elevated levels of MMP-7 and TNC were detected in sclerotic glomeruli and fibrotic interstitium, culminating in the development of a validated serum biomarker panel for the early detection of DKD [17]. These applications exemplify how single-cell and single-nucleus transcriptomics can anchor noninvasive biomarkers to distinct pathological injuries.

#### 2.2.3. Transcriptomics Multi-Omics Association Analysis

Integrated multi-omics analysis represents a crucial methodological approach for the identification and validation of pathology-specific biomarkers. This approach operates within a closed-loop framework, beginning with the initial high-throughput screening of molecules related to DKD through proteomics, metabolomics, and transcriptomics [8]. Subsequently, the omics findings are precisely correlated with specific histopathological features of kidney biopsies, such as glomerulosclerosis and tubulointerstitial fibrosis [13]. The final step involves the prospective evaluation of candidate biomarkers to predict the progression of DKD, including declines in eGFR and the onset of ESRD, surpassing traditional indicators [30,31]. Several recent studies illustrate the efficacy of this strategy. For instance, the integration of transcriptomic and proteomic data has identified aldo-keto reductase family 1 member A1 (AKR1A1) as a potential biomarker associated with cellular dysfunction in DKD [13]. Single-cell transcriptomics, integrated with machine learning and Mendelian randomization, identified VCAN as a diagnostic marker that is dysregulated in both glomeruli and tubules [8]. Spatial transcriptomics, in conjunction with serum proteomics, localized MMP7 and TNC to sclerotic glomeruli and fibrotic interstitium, resulting in a three-protein panel (MMP7, SAA1, TNC) that has been validated in independent cohorts [17]. Urinary proteomics in biopsy-confirmed patients identified Serpin A4 and GGT1 as markers for distinguishing DKD from non-diabetic kidney disease [25]. Collectively, these examples illustrate how multi-omics discoveries can be precisely associated with specific renal lesions, offering a histopathologically grounded foundation for noninvasive biomarkers. Subsequent prospective validation of these biomarkers for clinical endpoints, such as eGFR decline and ESRD, and their incremental value over UACR have been documented [30,31,32].

To realize this pathway, it is essential to integrate two distinct types of analyses. The first involves cross-scale data integration, which amalgamates transcriptomic, proteomic, and metabolomic data with pathological phenotypes. This approach facilitates the analysis of molecular associations between biopsy tissues and corresponding body fluids (such as blood and urine), thereby enabling the identification of reliable biomarkers that not only reflect tissue lesions but are also amenable to noninvasive detection [13]. The second complementary approach entails algorithm-optimized screening through the application of machine learning techniques, including LASSO regression and support vector machines. These algorithms enhance the efficiency and accuracy of selecting effective biomarker combinations from high-dimensional omics data [8,33].

### 2.3. Technical Integration Pathway

**Figure 2:**
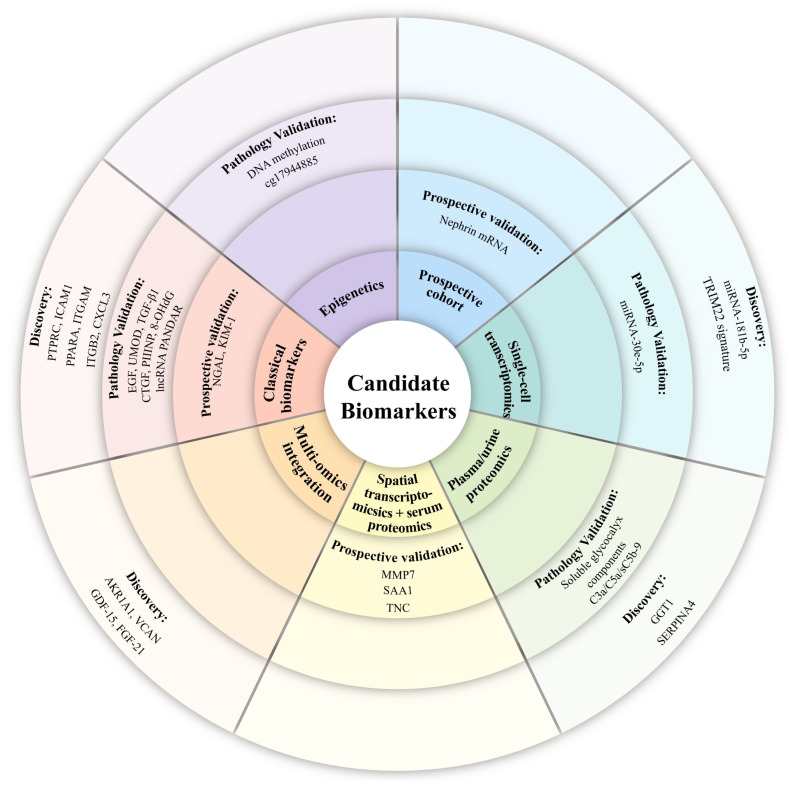
Development of biomarkers for diabetic kidney disease. Integrated view of classical and emerging biomarkers across epigenomics, transcriptomics, proteomics, and multi-omics for kidney diseases. NGAL, neutrophil gelatinase-associated lipocalin; KIM-1, kidney injury molecule-1; EGF, epidermal growth factor; UMOD, uromodulin; TGF-β1, transforming growth factor β1; CTGF, connective tissue growth factor; PIIINP, procollagen type III N-terminal propeptide; 8-OHdG, 8-hydroxy-2′-deoxyguanosine; lncRNA, long non-coding RNA; PANDAR, promoter of CDKN1A antisense DNA damage activated RNA; PTPRC, protein tyrosine phosphatase receptor type C; ICAM1, intercellular adhesion molecule 1; PPARA, peroxisome proliferator-activated receptor alpha; ITGAM, integrin alpha-M; ITGB2, integrin beta 2; CXCL3, chemokine (C-X-C Motif) ligand 3; AKR1A1, aldo-keto reductase family 1 member A1; VCAN, versican; GDF-15, growth differentiation factor-15; FGF-21, fibroblast growth factor-21; MMP7, matrix metalloproteinase 7; SAA1, serum amyloid A1; TNC, tenascin C; SERPINA4, Serpin A4; GGT1, gamma-glutamyltransferase 1; TRIM22, tripartite motif-containing protein 22.

From an integrated multi-omics perspective, candidate renal biomarkers are systematically categorized into seven distinct derivation groups, encompassing both conventional clinical markers and novel molecules derived from multi-omics analyses. Figure 2 illustrates the technical framework grounded in the overarching research logic of pathology-anchored biomarkers. Traditional biomarkers are aligned with routinely utilized clinical indicators that have historically supported the detection of tubular injury in clinical practice. Epigenetic methodologies identify aberrant DNA methylation loci as molecular signatures indicative of early renal pathological changes. In contrast, prospective cohort studies corroborate transcript-level markers linked to the structural integrity of glomeruli. Advanced sequencing technologies, such as single-cell transcriptomics, reveal non-coding RNA-based molecular signatures with enhanced early diagnostic potential. Additionally, plasma and urine proteomics identify circulating and urinary protein markers that signal renal epithelial barrier dysfunction. The integration of spatial transcriptomics with serum proteomics enables the identification of spatially specific circulating biomarkers that reflect renal inflammation and fibrosis progression [17]. Furthermore, the application of multi-omics integration addresses the limitations associated with single-platform analyses, thereby facilitating the identification of robust cross-layer candidate markers [17]. This comprehensive classification framework effectively distinguishes traditional clinical biomarkers from novel candidates derived from multi-omics approaches, offering a well-structured theoretical foundation for the stratified screening and clinical translation of diagnostic and prognostic markers across various kidney diseases. This figure provides a qualitative classification of the renal tissue lesion types associated with the pathology-anchored noninvasive biomarkers discussed in this review, as well as their potential applications. The different sections of the figure represent major lesion types, including glomerular lesions such as podocyte injury and endothelial glycocalyx injury; tubulointerstitial lesions such as tubular injury and reduced distal tubular function; renal fibrosis, exemplified by interstitial fibrosis and extracellular matrix deposition; and inflammation or metabolic reprogramming, indicated by inflammatory factors and molecules related to mitochondrial dysfunction [14,18,34]. The outer ring of the figure denotes potential applications of these biomarkers, encompassing early diagnosis, prognostic assessment, and therapeutic response monitoring [4,18]. It should be noted that, to date, no pathology-anchored biomarker has been officially approved for the independent early diagnosis of DKD, and the classifications depicted in the figure reflect only the current state of research [4].

It should be noted that biomarkers may simultaneously possess a solid pathological basis and clinical translational potential [14,22]. In the initial stage, pathology is derived from patients with DKD who present clinical indications warranting a biopsy, as opposed to those in the early stages of the disease. The primary objective at this stage is to identify molecules specific to the pathology [35]. Subsequently, the second stage involves validating the early diagnostic utility of these biomarkers in independent high-risk populations, where traditional indicators remain within normal ranges [36]. Although this stage does not depend on pathological findings, it necessitates long-term follow-up. This approach underpins the rationale for utilizing pathology anchoring in the early diagnosis process.

## 3. Application of Pathology-Anchored Biomarkers in DKD

### 3.1. Biomarkers Anchored to Glomerular Lesions

Glomerular lesions represent fundamental pathological alterations in DKD, encompassing podocyte injury, endothelial damage, mesangial expansion, and nodular sclerosis, such as Kimmelstiel–Wilson nodules. The pathological classification criteria established by Tervaert et al. offer a histological foundation for biomarker research [34].

#### 3.1.1. Biomarkers Related to Podocyte Injury in DKD

Podocytes play a crucial role in the glomerular filtration barrier, and early podocyte injury and detachment are pivotal initiating events in DKD. Pathology-based studies have elucidated the molecular underpinnings of such injuries and have identified several potential noninvasive biomarkers. Abnormalities in essential podocyte slit diaphragm proteins, such as nephrin and podocin, are central to glomerular injury in DKD. Urinary nephrin mRNA levels exhibit a negative correlation with podocyte density in kidney biopsy specimens, and their elevation can precede the onset of microalbuminuria. The prognostic value of this biomarker has been corroborated by an independent prospective cohort study with 2–3 years of follow-up, wherein normoalbuminuric patients with elevated baseline urinary nephrin mRNA levels demonstrated a significantly increased risk of progressing to microalbuminuria, with a hazard ratio of approximately 2.9 [16]. Consequently, this biomarker has undergone preliminary two-stage validation and stands as one of the pathology-anchored biomarkers nearest to clinical application. It has the capability not only to diagnose existing subclinical pathological injury but also to predict future clinical events, thereby providing a rationale for its use in early diagnosis [4,37].

In addition to protein and mRNA biomarkers, advancements in technologies such as single-cell omics have broadened the spectrum of biomarkers associated with podocyte injury. Specific microRNAs (miRNAs) have been identified as being closely linked to podocyte injury and the progression of DKD. For instance, miR-181b-5p is consistently downregulated in DKD mouse models; supplementing its mimic effectively alleviates proteinuria and renal pathological injuries, which identifies this microRNA as a promising therapeutic target for DKD [38]. Meanwhile, serum miR-30e-5p is significantly reduced in type 2 diabetic patients complicated with DKD, and its concentration is negatively correlated with the annual reduction rate of eGFR, robustly supporting the clinical potential of miR-30e-5p as a novel prognostic biomarker to monitor DKD disease progression [39].

Current research endeavors to identify noninvasive diagnostic indicators that surpass traditional albuminuria in specificity and temporal sensitivity by precisely correlating podocyte-related molecules in urine—such as specific mRNAs, miRNAs, and protein fragments—with pathological changes in renal tissue. These biomarkers hold potential for facilitating early detection, progression assessment, and targeted intervention in DKD.

#### 3.1.2. Biomarkers Related to Endothelial Glycocalyx Injury in DKD

Injury to the glomerular endothelial glycocalyx represents an early characteristic of microvascular lesions in DKD. Empirical investigations have substantiated that specific biomarkers can accurately reflect glomerular endothelial dysfunction by demonstrating correlations between soluble glycocalyx components or endothelial activation markers in plasma and endothelial lesion scores in renal tissue. Proteomic analyses have further identified aberrant expression of complement proteins, such as C3, and proteins involved in the coagulation pathway, such as the fibrinogen gamma chain, in the plasma of patients with DKD. Increased deposition of C3a, C5a, and the membrane attack complex C5b-9 has been observed in the renal tissues of DKD patients, with urinary levels correlating with the extent of glomerular complement deposition. This suggests that complement activation plays a role in the progression of DKD [40,41]. These alterations in inflammation and coagulation, which are closely linked to endothelial damage and microvascular lesions, represent a potential source of noninvasive biomarkers and may offer insights into detecting endothelial injury prior to the onset of microalbuminuria [18,42,43].

#### 3.1.3. Biomarkers Related to Mesangial Matrix Expansion in DKD

Mesangial expansion represents an early structural alteration commonly observed in DKD. The transforming growth factor β (TGF-β) superfamily, which includes TGF-β1, TGF-β2, and TGF-β3, is pivotal in the development of this lesion and the subsequent progression to renal fibrosis. Among these isoforms, TGF-β1 has been the most extensively investigated, particularly for its role in promoting the accumulation of extracellular matrix components [18]. Non-coding RNAs (ncRNAs) have emerged as significant regulators within this pathway. Both miRNAs and long non-coding RNAs (lncRNAs) are involved in modulating TGF-β signaling, thereby influencing mesangial matrix metabolism. For instance, the dysregulation of several miRNAs, including miR-21, miR-29, and miR-192, has been implicated in the progression of mesangial expansion through their targeting of key elements within the TGF-β/Smad pathway [44]. Furthermore, serum levels of the long non-coding RNA promoter of CDKN1A antisense DNA damage activated RNA (PANDAR) have shown a positive correlation with proteinuria and a negative correlation with glomerular filtration rate in patients with DKD, suggesting its potential utility as a biomarker for disease progression [44,45]. Related Biomarkers anchored to glomerular lesions have been summarized in Table 1.

To evaluate the strength of evidence for these biomarkers, we stratify all candidates presented in Table 1, Table 2, Table 3 and Table 4 into three tiers based on the completeness of their research workflows. The highest tier comprises markers with prospective validation and verified clinical outcomes, which have completed both Stage 1 pathological anchoring and Stage 2 prospective follow-up verification in an independent normoalbuminuric cohort; representative molecules in this category include urinary nephrin mRNA, NGAL, and KIM-1. The second tier consists of markers with pathology validation confirmed by biopsy, demonstrating a definite quantitative correlation with renal histological lesions but lacking large-scale prospective cohort testing; these include miRNA-30e-5p, PANDAR, EGF, UMOD, TGF-β1, PIIINP, 8-OHdG, and DNA cg17944885 methylation. The third tier encompasses discovery-stage candidates derived solely from exploratory transcriptomic, proteomic, or metabolomic screening, without direct histological validation or prospective clinical verification; representative molecules include TRIM22, AKR1A1, CXCL3, VCAN, GDF-15, FGF-21, PTPRC, ITGAM, and ITGB2. Notably, SBP-1 and PKM2, which are referenced in peer literature, are general early biomarkers for acute kidney injury and chronic kidney disease rather than DKD-specific pathology-anchored markers. Therefore, they are cited only briefly as cross-disease comparative examples and are not core subjects of this review.

### 3.2. Biomarkers Anchored to Tubulointerstitial Lesions

Recent research substantiated that tubulointerstitial injury is pivotal in the progression of DKD and may serve as a more accurate predictor of renal function decline than glomerular lesions [46,47]. Beyond traditional markers such as neutrophil gelatinase-associated lipocalin (NGAL) and kidney injury molecule-1 (KIM-1), injury-specific molecular signatures of renal tubular epithelial cells, identified through single-cell transcriptomics encompassing altered microRNA clusters including miR-30e-5p and miR-181b-5p, as well as dysregulated long noncoding RNAs and tubular-derived stress-related coding genes, have been directly associated with acute or chronic tubular injury scores in kidney biopsies [4,48]. From a metabolic standpoint, urinary metabolomics have demonstrated that a combined model consisting of tyramine and phenylalanyl-proline possess a significant diagnostic value in differentiating DKD from diabetes alone [49]. Similarly, urine metabolomic profiling reveals stage-specific metabolic disturbances in the progression of DKD, highlighting significant dysregulation in biotin metabolism and taurine-hypotaurine metabolism, with biotin and taurine serving as key regulatory molecules. Additionally, five differential metabolites—hypoxanthine, N-acetyl-DL-histidine, cortisol, tetrahydrobiopterin and L-kynurenine—are identified through two machine learning methodologies. These seven candidate biomarkers demonstrate a strong correlation with traditional clinical renal indices, such as the UACR and serum creatinine, and are validated for their potential in early DKD diagnosis [50]. Using a novel method named attenuated total reflection-Fourier transform infrared (ATR-FTIR) characterized the biochemical and metabolic alterations in the kidney and urine, in diabetic mouse experiments, progressive changes of urinary protein, lipid and carbohydrate spectral features correspond to worsening kidney damage. Combined with multivariate statistical and machine learning modeling, urinary spectral characteristics can accurately classify disease stages and reliably predict renal pathological injury degree [51].

Urinary EGF and UMOD reflect distal tubular functional reserve and repair capacity, independently predicting DKD progression risk in biopsy-confirmed cohorts. Theoretical evidence supports distal tubular injury as an early DKD event preceding microalbuminuria, yet their early warning efficacy in normoalbuminuric high-risk populations lacks Stage 2 prospective validation. Current Tervaert grading prioritizes glomerular scoring with simplified interstitial fibrosis/tubular atrophy (IFTA) evaluation, introducing systematic bias when assessing tubulointerstitial-predominant non-albuminuric DKD. Specific distal tubular reserve biomarkers are urgently required to compensate this evaluation gap [52]. Related Biomarkers anchored to tubular lesions have been summarized in Table 2.

**Table 2:** Biomarkers anchored to tubular lesions.

Pathological Target/ Category	Biomarker	Sample Source	Pathological Significance/ Clinical Association	Validation Stage	Evidence Level	Notes	References
Tubular injury	NGAL, KIM-1	Urine	Directly correlated with tubular injury scores; early warning indicators	Stage 1 + partial Stage 2	Pathology Validation + Partial Prospective	Some cohorts have prospective data	[4]
	Molecular signatures of injured tubular epithelial cells	Urine	Correlated with acute/chronic tubular injury scores in kidney biopsy	—	Discovery	Not specifically named	[4,48]
Distal tubular function	EGF, UMOD	Urine	Assess distal tubular reserve and repair capacity; predict DKD progression	Stage 1	Pathology Validation	Early value inferred; pathological anchor needs optimization	[53]

Abbreviation: NGAL, neutrophil gelatinase-associated lipocalin; KIM-1, kidney injury molecule-1; EGF, epidermal growth factor; UMOD, uromodulin; DKD, diabetic kidney disease.

### 3.3. Biomarkers Anchored to Renal Fibrosis

Renal interstitial fibrosis represents the fundamental pathological mechanism propelling DKD towards its terminal stage [54]. Research has identified circulating biomarkers linked to the severity of this condition from various mechanistic perspectives. TGF-β1 and connective tissue growth factor (CTGF) are pivotal mediators of fibrosis, with their urinary concentrations being associated with the extent of interstitial fibrosis [55,56]. Beyond protein biomarkers, products of extracellular matrix remodeling also hold significant value. In patients with DKD, serum levels of procollagen type III N-terminal propeptide (PIIINP) exhibit a positive correlation with the extent of renal interstitial fibrosis [57].

Oxidative stress and epigenetic modifications are integral components of the fibrotic process. Elevated serum levels of 8-hydroxy-2′-deoxyguanosine (8-OHdG), a biomarker indicative of oxidative DNA damage, have been significantly correlated with renal function decline in patients [58]. Furthermore, DNA methylation at specific CpG sites, such as cg17944885 located on chromosome 19p13.2 near the ZNF788 and ZNF20 genes, has been prospectively linked to an increased risk of ESRD. Higher methylation levels at this site have been shown to enhance predictive models for DKD progression beyond traditional clinical risk factors [31]. The integration of multidimensional biomarkers, encompassing tubular epithelial injury, metabolic disturbances, oxidative stress, and epigenetic changes, holds promise for more precise and earlier non-invasive Nephrin assessment and prognostic prediction of key pathological changes in DKD [18,59]. Related biomarkers anchored to renal fibrosis lesions have been summarized in Table 3.

**Table 3:** Biomarkers anchored to renal fibrosis lesions.

Pathological Target/ Category	Biomarker	Sample Source	Pathological Significance/ Clinical Association	Validation Stage	Evidence Level	Notes	References
Core fibrosis factors	TGF-β1, CTGF	Urine	Correlated with the degree of interstitial fibrosis	—	Pathology Validation	Further validation needed	[55,56]
Fibrosis biomarker	PIIINP	Serum	Positively correlated with renal interstitial fibrosis area	—	Pathology Validation	Further validation needed	[57]
Oxidative stress	8-OHdG	Serum	Reflects oxidative DNA damage; independently associated with renal function decline	—	Pathology Validation	Further validation needed	[58]
Epigenetics	DNA methylation, cg17944885 and other CpG sites	Blood	Predicts the risk of DKD progression to ESRD	Stage 2	Prospective Validation	Directly predicts ESRD, but pathological anchoring is relatively weak	[31]

Abbreviation: TGF-β1, Transforming growth factor β-1; CTGF, connective tissue growth facto; PIIINP, procollagen type III N-terminal propeptide; 8-OHdG, 8-hydroxy-2′-deoxyguanosine; DKD, diabetic kidney disease; ESRD, end-stage renal disease.

### 3.4. Metabolic Reprogramming-Related Biomarkers

Metabolic disorders are central to the pathogenesis of DKD. In individuals with DKD, there are significant alterations in branched-chain amino acids and sphingolipid metabolites, which are linked to insulin resistance and lipotoxicity [8]. In addition to disruptions in amino acid and lipid metabolism, mitochondrial dysfunction represents a critical aspect of the metabolic reprogramming observed in DKD. Two stress-responsive mitokines, growth differentiation factor-15 (GDF-15) and fibroblast growth factor-21 (FGF-21), are found at elevated levels in the circulation of patients with DKD, with their concentrations correlating with the extent of tubular mitochondrial injury [14]. GDF-15 is upregulated in response to mitochondrial stress and contributes to metabolic regulation through the integrated stress response pathway, whereas FGF-21 serves as a pivotal metabolic regulator, ensuring the maintenance of glucose and lipid homeostasis [14]. The HIF-1α signaling pathway represents an additional aspect of metabolic regulation, as HIF-1α facilitates the transition from oxidative phosphorylation to aerobic glycolysis under hypoxic conditions, thereby promoting metabolic reprogramming. Jiang et al. demonstrated that HIF-1α mitigates tubular injury through HO-1-mediated regulation of mitochondrial dynamics, offering mechanistic evidence for metabolism-related biomarkers [47]. Aldo-keto reductase family 1 member A1 (AKR1A1), identified via multi-omics screening, is part of the aldo-keto reductase superfamily involved in detoxification and glucose metabolism pathways; however, its pathology-associated characteristics require further validation [13]. The other candidate biomarkers listed in Table 4, such as protein tyrosine phosphatase receptor type C (PTPRC, also known as CD45), currently lack direct evidence connecting them to tissue metabolic pathways or pathological injury; their pathology-associated characteristics and potential metabolic significance remain to be elucidated. Related biomarkers anchored to metabolic reprogramming and other candidate biomarkers have been summarized in Table 4.

**Table 4:** Biomarkers related to metabolic reprogramming and other candidate biomarkers.

Pathological Target/ Category	Biomarker	Sample Source	Pathological Significance/ Clinical Association	Evidence Level	References
Mitochondrial dysfunction	GDF-15, FGF-21	Circulation	Correlated with the degree of tubular mitochondrial injury	Discovery	[14,40]
Inflammation/ immunity	PTPRC, ICAM1, PPARA	Blood/tissue, measurable in circulation	Fibrosis-related genes; PPARA is a DKD-specific candidate	Discovery	[60]
NETs-related	ITGAM, ITGB2	Blood	Related to neutrophil extracellular traps; reflects immune pathology	Discovery	[29]
Machine learning screening ^1^	CXCL3	Blood	Hub gene with diagnostic value	Discovery	[61]
Multi-omics Screening ^1^	AKR1A1	Blood	DKD biomarker identified by multi-omics	Discovery	[13]
Proteomics ^1^	Serpin A4, GGT1	Urine	Potential biomarkers for distinguishing DKD from non-DKD	Discovery	[25]

^1^ These candidate biomarkers are mostly screening results based on omics or bioinformatics and have not yet undergone rigorous renal tissue pathological association validation. Further studies are needed to confirm their pathology-anchored value. Abbreviation: GDF-15, growth differentiation factor-15; FGF-21, fibroblast growth factor-21; PTPRC, protein tyrosine phosphatase receptor type C; ICAM1, intercellular adhesion molecule 1; PPARA, peroxisome proliferator-activated receptor alpha; ITGAM, integrin alpha-M; ITGB2, integrin beta 2; CXCL3, chemokine (C-X-C Motif) ligand 3; AKR1A1, aldo-keto reductase family 1 member A1; GGT1, gamma-glutamyltransferase 1; DKD, diabetic kidney disease.

## 4. Clinical Validation and Application Potential of Biomarkers

Renal injury biomarkers anchored in pathology have demonstrated significant potential for multidimensional application in clinical validation. Their primary value resides in the early detection of disease, allowing for the identification of subclinical DKD in patients who exhibit normal eGFR and UACR. For instance, biomarkers such as urinary NGAL, KIM-1, and nephrin mRNA can predict the onset of disease several years prior to the manifestation of microalbuminuria. This provides a crucial supplementary diagnostic tool for patients with non-albuminuric DKD, who represent approximately 20–40% of cases [5,18,23,32].

In terms of differential diagnosis, biomarkers indicative of specific pathological processes, such as podocyte injury and complement activation, may facilitate the differentiation of DKD from non-diabetic kidney disease, which constitutes approximately 30–50% of kidney disease cases in patients with diabetes. Integrating these biomarkers with conventional indicators through joint modeling can substantially enhance the accuracy of differential diagnosis [5,8,34,62].

For the purposes of risk stratification and progression prediction, the correlation between biomarker levels and the extent of renal tissue injury serves as a foundational basis for their prognostic utility. Continuous monitoring of biomarker fluctuations, including baseline levels and annual changes, may offer superior predictive capability for ESRD risk compared to isolated measurements. In a study by Sharma et al., a predictive model was developed utilizing data from the Chronic Renal Insufficiency Cohort (CRIC), which included patients with chronic kidney disease stages 3–4. This model incorporated tumor necrosis factor receptor 1 (TNFR1), tumor necrosis factor receptor 2 (TNFR2), MMP-7, and eGFR. The model achieved a c-statistic of 0.931 for ESRD, demonstrating significantly improved predictive accuracy over models that rely solely on traditional indicators [26,37]. While this model is applicable for predicting progression risk in patients with existing renal function impairment, further independent validation is necessary to determine its accuracy in high-risk populations with normal UACR.

Moreover, a research framework that integrates multi-omics data with Mendelian randomization analysis, exemplified by a cohort–proteomics–Mendelian randomization triad, can elucidate the causal relationship between biomarkers such as angiogenin and VCAN and the progression of DKD. This approach offers genetic evidence and establishes new benchmarks for transitioning biomarkers from associative discovery to mechanistic interpretation and precision application [8,22,63,64,65].

## 5. Discussion

In this review, we systematically investigate the pathology-anchored approach and advocate for a two-step translational model. By synthesizing current evidence, we highlight that the clinical failure of many candidate biomarkers stems from a lack of mechanistic specificity and a disconnection from the underlying tissue injury. The proposed two-step framework—linking molecular signatures to specific histological lesions in biopsy cohorts (Step 1) prior to validation in non-invasive, high-risk populations (Step 2)—offers a logical solution to bridge the gap between discovery and clinical utility. While challenges regarding assay standardization and the inherent biases of extrapolating late-stage pathology to early disease remain, this pathology-centric approach provides a necessary roadmap for identifying biomarkers that are not merely statistical predictors, but true biological sentinels of incipient renal damage.

Current DKD biomarker research employs three mainstream frameworks: clinical outcome-driven discovery (convenient but lacks tissue specificity), omics-only discovery (broad but high false positives), and machine learning (accurate but poorly interpretable). In contrast to these methods, the pathology-anchored strategy provides direct mechanistic linkage to histological lesions, improving specificity; its limitation is biopsy dependence. Compared with previous biomarkers reviews, this review offers two innovations: lesion-anchored categorization by renal phenotypes, and a two-stage framework separating biopsy-based discovery from prospective validation. A three-tier evidence grading system distinguishes discovery, pathology-validated, and prospectively validated markers.

Despite its advantages in specificity, this framework is subject to two fundamental built-in biases that warrant thorough discussion. The first is the limitation regarding normoalbuminuric biopsies. Due to ethical constraints, there is no access to renal tissue from patients with normal UACR. This eliminates the possibility of direct pathological validation for candidate markers in true early-stage DKD populations, forcing all pathological anchors to rely on symptomatic, late-stage biopsy cohorts. The second issue is stage extrapolation bias, where biomarkers identified in advanced fibrotic DKD cannot be directly generalized to subclinical patients without longitudinal, multi-stage verification. Therefore, we emphasize that the two-stage pathology-anchored strategy presented here is not yet an established, mature clinical framework. Rather, it is a testable translational hypothesis that requires rigorous validation through large-scale, multi-center prospective cohorts.

A major challenge hindering the reliability of such research is the poor inter-cohort reproducibility, where many markers fail during external validation. This issue is primarily driven by three factors: small discovery cohorts with insufficient statistical power, a lack of standardized detection protocols, and significant population heterogeneity regarding diabetes duration, hypertension, medication use, and ethnicity. To address these reproducibility issues, it is essential to prioritize large-scale multi-center prospective cohorts and establish unified detection workflows with standardized reference intervals. Additionally, the field should shift from relying on single markers to utilizing multi-omics integrated panels. Combining spatial omics with serial biopsies in animal models will also be crucial to resolving the bias associated with extrapolating findings from late-stage to early DKD.

Finally, translating pathology-anchored biomarkers into clinical practice faces many barriers. A primary obstacle is that standardized reference intervals have not yet been established due to significant inter-laboratory variability. Furthermore, cost-effectiveness remains unproven; multi-omics platforms are currently too expensive for routine use, and targeted assays must demonstrate added value over existing metrics like UACR and eGFR.

In conclusion, resolving the reproducibility crisis in DKD biomarker research demands a synergistic approach combining rigorous study design with cutting-edge technology. This approach requires the prioritization of large-scale, multi-center prospective cohort studies and the establishment of unified assay protocols with standardized reference intervals to resolve the long-standing reproducibility crisis and population heterogeneity. Furthermore, future research must bridge the gap between clinical and pre-clinical findings by combining serial biopsy cohorts with spatial omics analyses of early DKD animal models. Ultimately, this progressive pathway—from standardized validation to mechanistic dissection—will not only rectify current discrepancies in biomarker verification but also catalyze the development of high-precision diagnostic tools, propelling DKD management into a new era of precision nephrology.

## Figures and Tables

**Table 1:** Biomarkers anchored to glomerular lesions.

Pathological Target/ Category	Biomarker	Sample Source	Pathological Significance/ Clinical Association	Validation Stage	Evidence Level	Notes	References
Podocyte injury	Nephrin mRNA	Urine	Negatively correlated with podocyte density in kidney biopsy; appears before microalbuminuria; predicts progression to microalbuminuria	Stage 1 + Stage 2	Prospective Validation	Prospective validation completed	[4,16]
	miRNA-30e-5p	Serum	Significantly decreased in DKD patients; negatively correlated with annual eGFR decline	Stage 1	Pathology Validation	Cross-sectional pathological association only	[39]
	miRNA-181b-5p	Animal model	Downregulated in DKD mouse models; supplementation reduces proteinuria	—	Discovery	Preclinical evidence	[38]
Endothelial glycocalyx injury	Soluble glycocalyx components and endothelial activation markers	Plasma	Correlated with endothelial lesion scores in kidney tissue; reflects endothelial dysfunction	—	Pathology Validation	Further validation needed	[18,42,43]
Endothelial complement activation	C3a, C5a, sC5b-9	Urine	Correlated with glomerular complement deposition; involved in DKD progression	—	Pathology Validation	Further validation needed	[40,41]
	lncRNA PANDAR	Serum	Positively correlated with proteinuria and negatively correlated with eGFR	—	Pathology Validation	Reflects mesangial lesions	[44,45]

Abbreviation: DKD, diabetic kidney disease; miRNA, microRNA; lncRNA, long non-coding RNA; PANDAR, promoter of CDKN1A antisense DNA damage activated RNA; eGFR, estimated glomerular filtration rate.

## Data Availability

No new data were created or analyzed in this study. Data sharing is not applicable.

## Abbreviations

The following abbreviations are used in this manuscript:

DKD	Diabetic kidney disease
ESRD	End-stage renal disease
UACR	Urinary albumin-to-creatinine ratio
eGFR	Estimated glomerular filtration rate
MMP-7	Matrix metalloproteinase 7
SAA1	Serum amyloid A1
TNC	Tenascin C
VCAN	Versican
TRIM22	Tripartite motif-containing protein 22
COL1A1	Collagen type I alpha 1
COL1A2	Collagen type I alpha 2
FN1	fibronectin 1
LUM	Lumican
scRNA-seq	Single-cell RNA sequencing
snRNA-seq	Single-nucleus RNA sequencing
miRNAs	MicroRNAs
TGF-β	Transforming growth factor β
ncRNAs	Non-coding RNAs
lncRNAs	Long non-coding RNAs
PANDAR	promoter of CDKN1A antisense DNA damage activated RNA
NGAL	Neutrophil gelatinase-associated lipocalin
KIM-1	Kidney injury molecule-1
EGF	Epidermal growth factor
CTGF	Connective tissue growth factor
UMOD	Uromodulin
PIIINP	Procollagen type III N-terminal propeptide
ATR-FTIR	Attenuated total reflection-Fourier transform infrared
8-OHdG	8-hydroxy-2′-deoxyguanosine
GDF-15	Growth differentiation factor-15
FGF-21	Fibroblast growth factor-21
AKR1A1	Aldo-keto reductase family 1 member A1
PTPRC	Protein tyrosine phosphatase receptor type C
ICAM1	Intercellular adhesion molecule 1
PPARA	Peroxisome proliferator-activated receptor alpha
ITGAM	Integrin alpha-M
ITGB2	Integrin beta 2
CXCL3	Chemokine (C-X-C Motif) ligand 3
GGT1	Gamma-glutamyltransferase 1
CRIC	Chronic Renal Insufficiency Cohort
TNFR1	Tumor necrosis factor receptor 1
TNFR2	Tumor necrosis factor receptor 2
